# Production of the Bioactive Compounds Violacein and Indolmycin Is Conditional in a *maeA* Mutant of *Pseudoalteromonas luteoviolacea* S4054 Lacking the Malic Enzyme

**DOI:** 10.3389/fmicb.2016.01461

**Published:** 2016-09-16

**Authors:** Mariane S. Thøgersen, Marina W. Delpin, Jette Melchiorsen, Mogens Kilstrup, Maria Månsson, Boyke Bunk, Cathrin Spröer, Jörg Overmann, Kristian F. Nielsen, Lone Gram

**Affiliations:** ^1^Department of Biotechnology and Biomedicine, Technical University of Denmark Kongens Lyngby, Denmark; ^2^Department of Microbial Ecology and Diversity Research, Leibniz Institute DSMZ–German Collection of Microorganisms and Cell Cultures – Partner Site Hannover-Braunschweig, German Centre for Infection ResearchBraunschweig, Germany

**Keywords:** *Pseudoalteromonas luteoviolacea*, indolmycin, violacein, conditional expression, antibacterial activity

## Abstract

It has previously been reported that some strains of the marine bacterium *Pseudoalteromonas luteoviolacea* produce the purple bioactive pigment violacein as well as the antibiotic compound indolmycin, hitherto only found in *Streptomyces*. The purpose of the present study was to determine the relative role of each of these two compounds as antibacterial compounds in *P. luteoviolacea* S4054. Using Tn*10* transposon mutagenesis, a mutant strain that was significantly reduced in violacein production in mannose-containing substrates was created. Full genome analyses revealed that the *vio*-biosynthetic gene cluster was not interrupted by the transposon; instead the insertion was located to the *maeA* gene encoding the malic enzyme. Supernatant of the mutant strain inhibited *Vibrio anguillarum* and *Staphylococcus aureus* in well diffusion assays and in MIC assays at the same level as the wild type strain. The mutant strain killed *V. anguillarum* in co-culture experiments as efficiently as the wild type. Using UHPLC-UV/Vis analyses, we quantified violacein and indolmycin, and the mutant strain only produced 7–10% the amount of violacein compared to the wild type strain. In contrast, the amount of indolmycin produced by the mutant strain was about 300% that of the wild type. Since inhibition of *V. anguillarum* and *S. aureus* by the mutant strain was similar to that of the wild type, it is concluded that violacein is not the major antibacterial compound in *P. luteoviolacea*. We furthermore propose that production of violacein and indolmycin may be metabolically linked and that yet unidentified antibacterial compound(s) may be play a role in the antibacterial activity of *P. luteoviolacea*.

## Introduction

The production of bioactive secondary metabolites is an essential defense mechanism and/or competitive strategy for many bacterial species. Violacein, a purple bisindole metabolite, has been extensively studied and has been reported to be potently antibacterial, antiviral, anti-tumor, antiprotozoal, and antiparasitic (Balibar and Walsh, 2006; Durán et al., 2007). It is produced by members of the Beta- and Gamma-proteobacteria, and has enabled species to occupy new niches. The skin-dwelling *Janthinobacterium lividum*, for example, shares a mutualism with its amphibian host, synthesizing violacein that provides antifungal protection to its host (Brucker et al., 2008). In the marine environment, violacein-producing species of the *Pseudoalteromonas* genus have been isolated from marine sponges (Yang et al., 2007), from biofilms on bivalve shells (Gillan et al., 1998), and from other biotic surfaces such as the alga *Ulva australis* (Rao et al., 2007). These niches are typically high in bacterial density, however, it is not known if or how effective violacein is in mediating bacteria–bacteria-interactions.

The focus of this study is the purple marine bacterium *Pseudoalteromonas luteoviolacea* strain S4054, isolated from a seawater-immersed surface (Gram et al., 2010). Strains of *P. luteoviolacea* are distinctly purple due to the production of violacein (Gauthier, 1982; McCarthy et al., 1985) and have also been reported to synthesize an antimicrobial *L*-amino acid oxidase (Gómez et al., 2008), as well as the antibiotic compound pentabromopseudilin (Hanefeld et al., 1994). We previously detected violacein and pentabromopseudilin in strains of *P. luteoviolacea*, and we furthermore identified a non-violacein compound, indolmycin, from a culture of *P. luteoviolacea* S4054 where pentabromopseudilin was not detected (Månsson et al., 2010; Vynne et al., 2012). Both violacein and indolmycin are based on an indole scaffold, biosynthetically derived from L-tryptophan (Hurdle et al., 2004; Balibar and Walsh, 2006).

Indolmycin is a secondary metabolite with antibacterial properties which was first isolated from *Streptomyces griseus* (Hornemann et al., 1971). To date, indolmycin biosynthesis has not been reported in bacteria outside of the phylum Actinobacteria except for a few strains of *P. luteoviolacea* (Månsson et al., 2010; Vynne et al., 2011, 2012). Indolmycin acts as an antimicrobial compound due to its tryptophanyl-tRNA synthase inhibition activity (Werner et al., 1976), ceasing bacterial protein synthesis (Oliva et al., 2003). It is bacteriostatic against *Staphylococcus aureus*, and has been tested as a potential topical agent for use against both methicillin-resistant and vancomycin-intermediate *S. aureus* (Hurdle et al., 2004). However, indolmycin also has a bactericidal effect on Gram negative *Helicobacter pylori* (Kanamaru et al., 2001), and it has therefore been speculated that this compound has different modes of action on bacteria (Hurdle et al., 2004).

Vynne et al. (2011) clustered four strains of *Pseudo alteromonas luteoviolacea* based on the detection of their secondary metabolites by HPLC-UV/Vis. Two strains (S4054 with S4047) produced both violacein and indolmycin and two strains (S4060 with S2607) produced violacein and pentabromopseudilin (Vynne et al., 2011). Whilst violacein is difficult to dissolve in water and accumulates in the bacterial membrane (Konzen et al., 2006), both pentabromopseudilin and indolmycin are highly water soluble and may each serve the same role in the four strains. Månsson et al. (2016) analyzed the metabolome of 13 strains of *P. luteoviolacea*, and found a large variety in biochemical potential within each strain (Månsson et al., 2016). Also, a gene cluster consisting of 12 genes was identified as the potential indolmycin biosynthetic gene cluster. This adds to the nine genes identified by Du et al. (2015) encoding indolmycin in *Streptomyces griseus*, including two genes identified as homologues of the quorum sensing genes *luxI* and *luxR* which were not observed in *S. griseus* (Månsson et al., 2016).

In the present study, we sought to determine the importance of violacein in the antibacterial activity of *P. luteoviolacea* S4054. We initially attempted a targeted gene deletion approach but were unable to manipulate *P. luteoviolacea* in this manner and we therefore used a random mutagenesis approach, creating a mutant with negligible violacein production. Additionally, methodology was established to simultaneously quantify violacein and indolmycin against commercial standards to gain an accurate insight into the levels of each chemical compound being produced by *P. luteoviolacea* S4054 and mutant strains hereof.

## Materials and Methods

### Bacterial Strains, Media, and Growth Conditions

*Pseudoalteromonas luteoviolacea* S4054 was isolated during the Danish research expedition “Galathea 3” (Gram et al., 2010). Strain S4054-2 is a spontaneous streptomycin resistant (Sm^R^) mutant of S4054, which was isolated after overnight incubation at 25°C of strain S4054 on Marine Agar (MA; Difco 2216) supplemented with 200 μg ml^-1^ streptomycin. Strain S4054-2-49 is a transposon (Tn) mutant generated from strain S4054-2, containing a single random insertion of miniTn*10:gfp:kan* (Stretton et al., 1998) in its genome. For broth cultures, all S4054-based strains were grown overnight at 25°C with shaking at 200 rpm. The liquid media used were: Marine Broth (MB; Difco 2216) and marine minimal medium (MMM) (Ostling et al., 1991) supplemented with 0.3% casamino acids (CAA; Difco 223050) and either 0.4% mannose or 0.4% glucose. Strain S4054-2 was furthermore supplemented with 200 μg ml^-1^ streptomycin, and strain S4054-2-49 was supplemented with 200 μg ml^-1^ streptomycin and 200 μg ml^-1^ kanamycin. All carbon sources and chemicals used in this study were obtained from Sigma-Aldrich (Saint Louis, MO, USA) unless otherwise stated.

Target organisms for antibacterial susceptibility testing were *Vibrio anguillarum* 90-11-287 (Skov et al., 1995) and *V. anguillarum* NB10 (Milton et al., 1992) grown in MB or Tryptone Soy Broth (TSB; Oxoid CM129) or on MA or Tryptone Soy Agar (TSA; Oxoid CM131) and *Staphylococcus aureus* 8325 (Novick, 1967) grown in Luria Broth (LB) or on Luria Agar (LB agar) (Oxoid CM996B). All target strains were grown at 25°C. For co-cultivation experiments, we used a variant of *V. anguillarum* NB10 that was tagged with chloramphenicol resistance (Cm^R^) by insertion of plasmid pNQFlaC4-gfp27 (*cat, gfp*) into an intergenic region on the chromosome, *V. anguillarum* NB10 (Cm^R^), kindly provided by D. Milton, University of Umeå.

Strains used for transposon mutagenesis were *Escherichia coli* DH5α(pNJ5000) and *E. coli* SM10AAApir(pLOF::miniTn*10:gfp:kan*) (Stretton et al., 1998). *E. coli* strains were grown at 37°C on LB agar or in LB with shaking at 200 rpm. Streptomycin (200 μg ml^-1^), kanamycin (200 μg ml^-1^), tetracycline (10 μg ml^-1^), and ampicillin (100 μg ml^-1^) were used for selection.

To examine the conditional expression of violacein, Na-pyruvate and L-Tryptophan was added to the MMM medium containing CAA and mannose to a final concentration of 11 mg/ml and 1 mg/ml, respectively.

### Transposon Mutagenesis and Selection of Mutants

A violacein-negative mutant of *P. luteoviolacea* was created by conjugation with an *E. coli* donor carrying the pLOF::miniTn*10:gfp:kan* plasmid. This plasmid is readily mobilized from *E. coli* to *P. luteoviolacea* but since the replication of the plasmid is dependent upon a non-conjugative helper plasmid (pNJ5000), stable kanamycin resistant (Km^R^) transconjugants can only be obtained if the miniTn*10* transposes into the host chromosome (Stretton et al., 1998). Selection of Km^R^ *P. luteoviolacea* transposon mutants required that the *E. coli* donor cells could be selected against. Therefore a spontaneous Sm^R^ derivative of strain S4054 was selected on plates containing streptomycin. One such mutant, S4054-2 was shown to grow similar to the parental strain S4054 on marine agar (MA) as well as in MMM supplemented with casamino acids (CAA).

Triparental conjugation of *P. luteoviolacea* S4054-2, *E. coli* DH5α(pNJ5000), and *E. coli* SM10AAApir(pLOF::miniTn*10:gfp:kan*) was performed on a HATF filter membrane (0.45 μm; Merck Millipore Co., Darmstadt, Germany) placed on MA supplemented with 0.4% glucose. Briefly, all three strains were grown for 18 h in their corresponding medium and optimal temperature. Fifty microliters of each strain were mixed and pipetted onto the HATF membrane. After overnight incubation at 25°C, growth on the filter was resuspended in 1 ml MB and 10 × 100 μl volumes were plated onto MA supplemented with streptomycin and kanamycin. After overnight incubation at 25°C, 50 colonies that grew were re-streaked onto MA supplemented with streptomycin and kanamycin. Mutant strains were selected based on reduced purple pigmentation as compared to strain S4054-2. Additionally, mutants were screened for green fluorescent protein (GFP) expression (using an Olympus BX50 microscope fitted with an epifluorescence filter; 488 nm excitation, 520 nm emission), indicative of the insertion of the promoterless *gfp* transposon in the correct orientation under the transcriptional control of the promoter of the interrupted gene.

To confirm the presence of the miniTn*10:gfp:kan* in strain S4054-2-49, a 1.3 kb region of the cassette was amplified by PCR with HotStarTaq DNA polymerase (Qiagen, Venlo, Netherlands), performed according to the manufacturer’s instructions, using 0.2 μM of each primer kmseq-F and gfpsfiI-F (Stretton et al., 1998). PCR cycling conditions: 94°C for 3 min; (94°C for 30 s, 53°C for 30 s, 72°C for 60 s) × 30; 72°C for 10 min. PCR products were analysed and visualized by gel electrophoresis using 1% TAE agarose gels (Promega, Madison, WI, USA).

### PacBio Library Preparation and Sequencing

To determine the site of the Tn insertion in strain S4054-2-49 and to detect other potentially random mutations that might affect the phenotypes of the mutant strains, *P. luteoviolacea* S4054, S4054-2, and S4054-2-49 were whole genome sequenced. Genomic DNA from the three strains was purified using the phenol/chloroform/isoamyl alcohol protocol described by Wilson (2001). SMRTbell^®^ template library was prepared according to the instructions from Pacific Biosciences Inc. (Menlo Park, CA, USA) following the Procedure and Checklist – 20 kb Template Preparation Using BluePippin^®^ Size-Selection System. Briefly, for preparation of 15 kb libraries 5 μg genomic DNA were end-repaired and ligated overnight to hairpin adapters applying components from the DNA/Polymerase Binding Kit P6 (Pacific Biosciences Inc.). Reactions were carried out according to the manufacturer’s instructions. BluePippin^®^ Size-Selection to 10 kb was performed according to the manufacturer’s instructions (Sage Science, Beverly, MA, USA). Conditions for annealing of sequencing primers and binding of polymerase to purified SMRTbell^®^ template were assessed with the Calculator in RS Remote (Pacific Biosciences Inc.). SMRT sequencing was carried out on the PacBio RSII (Pacific Biosciences Inc.) taking one 240-min movie for each SMRT cell. One SMRT cell per strain was run. In total, for strain S4054 87,476 reads (mean read length 9,065 bp), for strain S4054-2 74,109 reads (mean read length 9,226 bp), and for strain S4054-2-49 85,558 reads (mean read length 11,797 bp) were obtained.

### Genome Assembly, Error Correction, and Annotation

Data from each SMRT Cell was assembled independently using the “RS_HGAP_Assembly.3” protocol included in SMRTPortal version 2.3.0 using default parameters. Each assembly revealed two circular chromosomes, but no plasmids. Validity of each assembly was checked using the “RS_Bridgemapper.1” protocol. Each replicon was circularized independently, particularly artificial redundancies at the ends of the contigs were removed and the two chromosomes were additionally adjusted to *dnaA* or *tus* as the first gene, respectively (Médigue et al., 2005). Finally, each genome was error-corrected by a mapping of Illumina reads onto finished genomes using BWA (Li and Durbin, 2009) with subsequent variant calling using VarScan (Koboldt et al., 2012). A consensus concordance of QV60 could be confirmed for all of the three genomes. Finally, all genomes were annotated using Prokka 1.8 (Seemann, 2014) and RAST (Aziz et al., 2008), and aligned using the genome alignment software MAUVE 2.4.0 (Darling et al., 2004). Single genes of interest were translated to amino acid sequences and aligned using CLC Main Workbench 7.6.4 and used in homology searches using blastp (Basic Local Alignment Search Tool^1^). The genomes have been deposited in NCBI GenBank under Accession Numbers CP015411-CP015416.

The genomes were analyzed for putative biosynthetic gene clusters using AntiSMASH and the ClusterFinder algorithm (Medema et al., 2011; Weber et al., 2015).

### Screening for Antibacterial Activity

The ability of *P. luteoviolacea* S4054, S4054-2, and S4054-2-49 to inhibit the growth of a target strain (*V. anguillarum* 90-11-287 and *S. aureus* 8325) seeded into agar substrates was tested as previously described (Hjelm et al., 2004; Gram et al., 2010). Briefly, the agar substrate contained 1.5% Instant Ocean (Aquarium Systems Inc., Sarrebourg, France), 0.3% CAA, 0.4% glucose and 1.2% agar (Oxoid Ltd., Hampshire, UK). For *S. aureus*, 1% peptone was added. Once cooled to 45°C and prior to pouring, 10 μl of target strain, which had been grown stagnant overnight at 25°C in its corresponding rich medium, was added to 20 ml agar substrate. For well diffusion agar assay, 5 mm diameter wells were punched into the agar surface and 50 μl of filter sterilized (0.2 μm filter; Merck Millipore Co.) culture supernatant was loaded into each well. The supernatants were produced by centrifugation and serial dilution of 24, 48, and 72 h cultures grown in MMM CAA mannose. After incubation, inhibition zones of the same size were identified for all three strains. Following 24 h incubation at 25°C, the diameter of the clearing zone produced due to inhibition of growth of the target strains from edge to edge of the zone, including the well, was measured. Each sample was tested in duplicate.

For minimum inhibition concentration (MIC) assay, target strains *V. anguillarum* 90-11-287 and *S. aureus* 8325 were cultured in MB and LB, respectively, and incubated stagnant at 25°C overnight. Strains S4054, S4054-2, and S4054-2-49 were cultured for 72 h at 25°C in MMM CAA mannose (no antibiotics), supernatants were sterile filtered (0.2 μm filters; Merck Millipore Co.), and tested in twofold dilutions in 100 μl target strain diluted to OD_600_ 0.01 in microtiter plates. Plates were incubated at 25°C overnight against *V. anguillarum* 90-11-287 and for 48 h against *S. aureus* 8325. All MICs were carried out with four replicates and repeated twice.

### *Vibrio anguillarum* Competition Experiments

*Vibrio anguillarum* NB10 (Cm^R^) was co-inoculated together with cultures of the *P. luteoviolacea* strains in MMM CAA mannose. All cultures were grown in a 50 ml volume of medium in a 250 ml Erlenmeyer flask in duplicate. Cultures of *V. anguillarum* NB10 (Cm^R^) and S4054, S4054-2 or S4054-2-49 were grown separately overnight in TSB (NB10, Cm^R^) and MMM CAA mannose (S4054 strains) and added to MMM CAA mannose to concentrations of 10^3^ (NB10, Cm^R^) and 10^6^ (S4054 strains) cells ml^-1^, respectively. Simultaneously, a MMM CAA mannose control culture was inoculated with 10^3^ CFU ml^-1^ *V. anguillarum* NB10 (Cm^R^). Samples were taken at regular intervals (0, 3, 6, 9, 12, and 24 h) and cell density was determined by dilution and surface plating. *Vibrio anguillarum* NB10 (Cm^R^) from the competition experiment was counted on TSA plates with 4 μg ml^-1^ chloramphinicol incubated overnight at 30°C, since S4054 did not grow on TSA with chloramphenicol nor at 30°C. Strains S4054-2 and S4054-2-49 were selected for on MA with streptomycin and incubated at 25°C, since *V. anguillarum* NB10 (Cm^R^) is streptomycin-sensitive.

### Quantification of Violacein and Indolmycin

Extraction of violacein and indolmycin for quantitative studies was tested using propan-2-ol, methanol, ethanol, and acetonitrile. Acetonitrile created a two-phase system of variable size due to the media salts and could not be used. Methanol and ethanol had to be added at 5 and 2 times the sample volume, respectively, to extract the violacein quantitatively. This resulted in a lower concentration of the analytes in the mixture and also a need to inject more of the mixture to maintain a reasonable UHPLC (Ultra High-Performance Liquid Chromatography) peak shape for di-demethylindolmycin. Overall, propan-2-ol provided the most accurate extraction, so 5 ml of bacterial culture (fresh or thawed from -20°C storage) were prepared for the quantification of violacein, indolmycin, and indolmycin precursors by the addition of an equal volume of propan-2-ol.

The mixture was inverted until homogeneous and placed in an ultrasonication bath for 30 min. Tubes were centrifuged (4,000 × *g*, 15 min) to pellet the biomass (colorless) from the extracted violacein, indolmycin and related compounds in solution. Seven hundred and fifty microlitres of supernatant was transferred to an autosampler vial. UHPLC-UV/Vis analysis was performed on a Dionex RSLC Ultimate 3000 system (Sunnyvale, CA, USA) using a 150 mm × 2 mm i.d., 2.6 μm Kinetex C_18_ column (Phenomenex, Torrance, CA, USA), running at 800 μl min^-1^ and 60°C using a binary linear solvent system of water (A) and acetonitrile (B) (both buffered with 50 μl l^-1^ trifluoroacetic acid; TFA). The gradient program was: *t* = 0, 15% B; *t* = 0.5 min 25% B; *t* = 6 min 65% B; and *t* = 7 100% B, keeping this for 1 min, then reverting to 15% in 1 min. A sample volume of 1.5 μl was injected.

Violacein (RT 2.76 min) was detected recording absorption at 578 ± 2 nm, and indolmycin (1.98 min), di-demethylindolmycin (RT 1.21 min), and the two mono-demethylindolmycin (1.60 and 1.64 min, integrated as one peak) by absorption at 219 ± 2 nm.

Quantification by external standard calibration using six different concentrations of violacein and indolmycin for the calibration (40, 20, 10, 5, 2, and 1 μM) resulted in a linear calibration curve with *R*^2^ of 0.9999 and 0.9997, respectively. Extraction efficiency was tested by extracting various batches of samples three consecutive times, showing an extraction efficiency of >97%. The di- and mono-demethylindolmycins were quantified using the indolmycin calibration curve, since these compounds have the same chromophore and thus molar extinction coefficient. Detection limits were 1 μM for violacein, and 10, 5, and 1 μM for di-demethylindolmycin, mono-demethylindolmycin, and indolmycin, respectively.

### Identification of Indolmycin-Related Compounds

Samples for HPLC-UV/MS analyses were prepared from 30 ml cultures in MB and MMM CAA glucose or mannose. The culture broth was extracted with Diaion HP20 (Sigma–Aldrich, St. Louis, MO, USA), which was subsequently filtered off and extracted with MeOH, while the pellet was extracted using ethyl acetate (EtOAc). Extracts of pellet and broth were pooled, filtered, and evaporated under N_2_ until dry. Samples were re-dissolved in methanol (MeOH) and transferred to autosampler vials for HPLC-UV/MS analysis. HPLC-UV/MS samples were analyzed using an Agilent 1100 HPLC system with a diode array detector (Agilent Technologies, Waldbronn, Germany) coupled to an LCT TOF mass spectrometer (Micromass, Manchester, UK) using a Z-spray ESI source. A Phenomenex Luna II C_18_ column (50 mm × 2 mm, 3 μm) was used for separation, applying an acetonitrile-water (20 mM formic acid) 0.3 ml min^-1^ gradient (15–100%) over 20 min at 40°C.

Indolmycin (2.1 mg) and di-demethylindolmycin (1.7 mg) were isolated from a 1 l culture of S4054-2-49 in MMM CAA mannose by EtOAc extraction (2 × 500 ml). The EtOAc phase was dried with Na_2_SO_4_, filtered, and evaporated until dry. The crude extract was separated using a Luna II C_18_ column (250 × 10 mm, 5 μm; Phenomenex) on a Gilson 322 liquid chromatograph with a 215 liquid handler (BioLab, Risskov, Denmark), with an automatic fraction collector, applying a gradient from 20 to 100% acetonitrile in water (buffered with 50 ppm TFA) over 20 min (5 ml min^-1^).

NMR spectra were recorded on a Varian Unity Inova 500 MHz spectrometer (Agilent) equipped with a 4 mm gHX Nano probe and with a spin rate of 2 kHz for all samples, using standard pulse sequences. The signals of the residual solvent protons and solvent carbons were used as internal references (*δ*_H_ 3.3 and *δ*_C_ 49.3 ppm for methanol-*d*_4_).

### Statistical Analyses

A two-sample two-tailed *t*-test (Zar, 1996) was used to compare the difference between two means of log transformed CFU ml^-1^ or concentrations of secondary metabolites.

## Results

### Selection of Mutants with Impaired Violacein Production from a miniTn*10* Transposon Library of *P. luteoviolacea* S4054-2

Violacein producing bacteria are easily detected by their deep violet color. Initially, we attempted to construct a deletion mutant in either of the *vio*-genes, but all attempts on site-directed mutagenesis failed. Instead, a random transposon insertion library of mutants was constructed to select for violacein-negative mutants of the violacein producing, Sm^R^ *P. luteoviolacea* strain S4054-2. The Sm^R^ mutant used for conjugation behaved similarly to the wild type in growth and well diffusion agar assays. Whole genome sequencing revealed that the Sm resistance could be attributed to two single nucleotide polymorphisms (SNPs), both of which lead to non-synonymous amino acid exchange: One occurred in the *rpsL* gene which encodes the 30S ribosomal protein S12 and the other in the *rsmG* gene encoding the ribosomal RNA small subunit methyltransferase G (**Table 1**). Mutations in these two genes are known to confer streptomycin resistance in, e.g., *E. coli*, *Campylobacter coli*, and *Mycobacterium* spp. (Timms et al., 1992; Springer et al., 2001; Olkkola et al., 2010) and lead to antibiotic overproduction in *Streptomyces coelicolor* and several species of Actinomycetes (Nishimura et al., 2007; Tanaka et al., 2009). Therefore, we deduce that the acquired streptomycin resistance in strain S4054-2 is a result of either of these two mutations.

**Table 1:** Mutations in *P. luteoviolacea* S4054 resulting in Sm^R^ strain S4054-2 and the *maeA* mutant S4054-2-49 based on WGS.

Strains	Type of mutation	Annotation of affected gene	Functional role	Effect of mutation
S4054 to S4054-2	A to G (aa: C to R, pos. 237)	*mreB*, Rod-shape determining protein MreB	Formation of the rod shape of cells	No phenotypic effect
	G to A (aa: P to S, pos. 91)	*rpsL*, 30S ribosomal protein S12	Confers streptomycin resistance in *E. coli*, high level	Streptomycin resistance
	C to T (aa: G to R, pos. 78)	*rsmG*, Ribosomal RNA small subunit methyltransferase G	Confers streptomycin resistance and antibiotic overproduction in Actinomycetes and Streptomycetes, low level	Streptomycin resistance and possibly indolmycin overproduction
S4054-2 to S4054-2-49	Insertion of Tn cassette	*maeA*, NAD-dependent malic enzyme	Conversion of malate to pyruvate	Interruption of *maeA*
	Insertion of 1 bp	*tonB*, Transport protein TonB	Cross-membrane uptake of specific substrates	Interruption of *tonB*


*Further mutations appeared from the genome alignments, but could either be attributed to sequencing errors or occurred in regions that could not be annotated. Tn cassette, transposon cassette; aa, amino acid; pos., refers to the amino acid position in the annotated protein sequence.*

**FIGURE 1:**
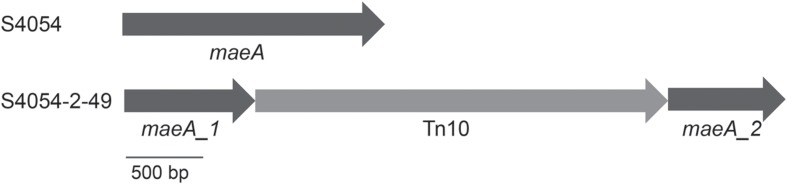
**Genetic arrangement of the Tn*10* insertion in the chromosome of *P. luteoviolacea* S4054-2.** The Tn*10* cassette is located between the N-terminal domain (*maeA_1*) and the NAD-binding domain (*maeA_2*) of the NAD-dependent malic enzyme encoding gene *maeA*.

Transposon mutagenesis of S4054-2 with *E. coli* DH5α/pLOF::miniTn*10:gfp:kan* resulted in a library of Sm^R^ and Km^R^ resistant S4054-2 *genX*::miniTn*10:gfp:kan* mutants, most of which were pigmented. Despite many repeated attempts only 50 mutants grew and one mutant S4054-2-49 showed a non-pigmented phenotype on MA plates containing streptomycin and kanamycin. PCR amplification of the *gfp-kan* junction in S4054-2-49 confirmed the presence of the miniTn*10:gfp:kan* and epifluorescence microscopy showed that the mutant, in contrast to S4054 and S4054-2, produced Gfp. Whole genome sequencing of S4054-2-49 showed that the miniTn*10* cassette had inserted into the *maeA* gene encoding a NAD-dependent malic enzyme that converts malate to pyruvate and CO_2_. The insertion sequence was located between the N-terminal domain and the NAD-binding domain of *maeA*, interrupting the production of a functional malic enzyme (**Figure 1**).

### Conditional Production of Violacein Pigmentation by the S4054-2 *maeA* Mutant

Selection of the *maeA* mutant was performed on solid MA medium, which contains sources of amino acids and vitamins (from peptone and yeast extract) as well as all required salts for S4054 to grow on. As detected during the selection of S4054-2-49 *maeA*, it showed a non-pigmented beige phenotype when grown on MA. After re-streaking the mutant, however, it was found that the colonies became purple after 2 days of growth at room temperature. This showed that the absence of the malic enzyme did not prevent the formation of violacein altogether, but rather delayed the production or redirected the metabolism away from violacein synthesis. Therefore we screened the mutant in a number of culturing conditions for the production of violacein pigment. The two parental strains, S4054 and S4054-2 (Sm^R^), produced pigments under all conditions tested, but the *maeA* mutant failed to produce pigment in tubes containing liquid MMM medium supplemented with CAA and mannose. Interestingly, growth on MMM agar plates resulted in fully pigmented S4054-2-49 *maeA* colonies, which showed that either the mutant required high oxygen tension for violacein synthesis (provided on plates), or that the agar substrate provided a required compound for biosynthesis. Since violacein is derived from the amino acid tryptophan, the effect of L-tryptophan addition to liquid MA was tested. Addition of Na-pyruvate was also tested because the absence of the malic enzyme in the mutant could lower the pyruvate production. Tryptophan did not promote purple pigmentation, but instead resulted in a more yellowish tint. Addition of pyruvate or pyruvate + tryptophan also resulted in beige and yellowish cultures, with no visible trace of purple color.

### Quantification of Violacein and Indolmycin in Wild Type and *maeA* Mutant

To obtain more quantitative evidence for the conditional production of violacein, we performed chemical analysis of 2-propanol extracts from culture samples after growth of S4054, S4054-2, and S4054-2-49 *maeA* mutant in liquid MMM CAA mannose medium. The *maeA* mutant was indeed deficient in violacein production as compared to the wild type (**Table 2**). The violacein concentration remained around 4 μM during 72 h of incubation in MMM CAA mannose, while the violacein concentration increased to 54 μM in the wild type culture, and to 80 μM in the S4054-2 Sm^R^ derivative.

**Table 2:** Antibacterial activity and mean violacein and indolmycin concentration (± standard error) produced by *P. luteoviolacea* S4054 and mutant strains in MMM CAA mannose.

Strain	Time (h)	Zone size of inhibition (mm)^∗^		Violacein (μM)^∗^	Indolmycin (μM)^∗^
				
		*V. anguillarum*	*S. aureus*		
S4054	24	23	28	43 ± 9	36 ± 2
	48	27	35	50 ± 6	46 ± 2
	72	28	31	54 ± 9	56 ± 2
S4054-2	24	19	26	74 ± 17	21 ± 1
	48	26	31	74 ± 10	28 ± 3
	72	26	32	83 ± 13	44 ± 2
S4054-2-49	24	20	26	4.1 ± 0.1	107 ± 3
	48	23	29	3.8 ± 0.05	141 ± 0.3
	72	26	31	4.0 ± 0.1	165 ± 0.7


*Sampling time (h; indicative of hours post-inoculation of the culture) is indicated. ^∗^*n* = 2.*

**FIGURE 2:**
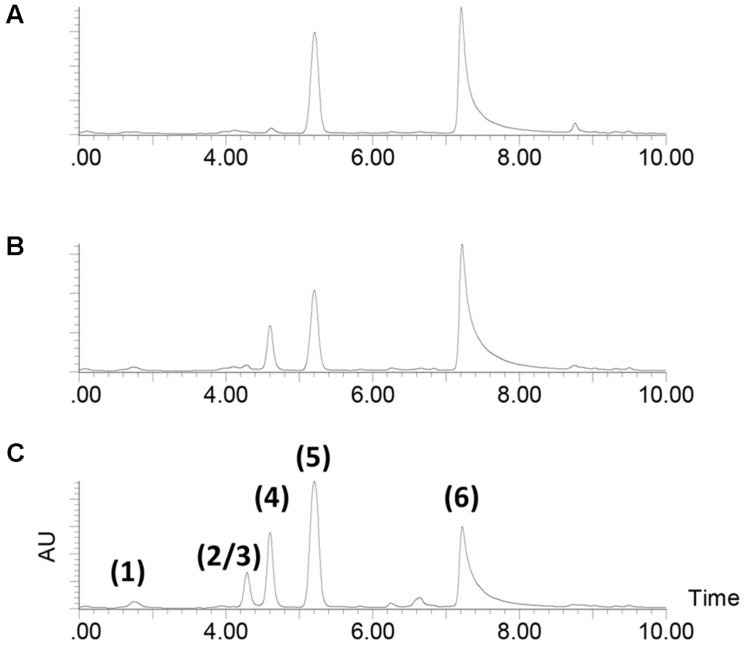
**HPLC-UV profiles of 72 h cultures grown in MMM CAA mannose.** **(A)** wild type strain S4054, **(B)** streptomycin resistant mutant strain S4054-2, **(C)** transposon mutant strain S4054-2-49. The identity of the peaks were determined by LC-HRMS: (1) N,C-didemethyl-indolmycin, (2/3) mono-N/C-demethyl-indolmycin, (4) indolmycenic acid, (5) indolmycin, and (6) violacein.

**FIGURE 3:**
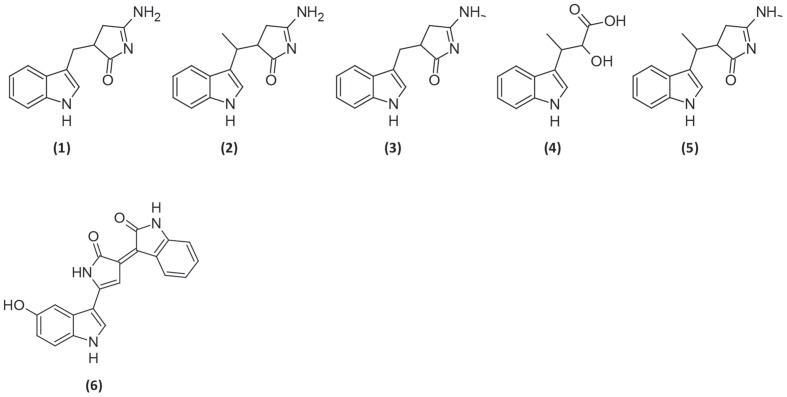
**Chemical structures of (1) N,C-didemethyl-indolmycin, (2/3) mono-N/C-demethyl-indolmycin, (4) indolmycenic acid, (5) indolmycin, and (6) violacein**.

During analysis of the mutant strains, a series of strong peaks was identified all of which were much higher than in the extracts from the wild type (**Figure 2**). The UHPLC-UV/MS profile of the Tn mutant S4054-2-49 *maeA* culture extract from MMM CAA mannose (**Figure 2C**) showed the expected peak for violacein (labeled 6 in **Figure 2C**) together with a series of compounds which could be identified as derivatives of indolmycin: di-demethylindolmycin (labeled 1 in **Figure 2C**), mono-N/C-demethylindolmycin (labeled 2 and 3), indolmycenic acid (labeled 4) (Hornemann et al., 1971), and indolmycin (labeled 5) (Speedie et al., 1975). Structures were validated by NMR for compounds 1, 5, and 6 (Månsson et al., 2010), while the remaining compounds were tentatively identified based on their retention time, accurate mass and the deduced molecular formulas together with their UV/Vis characteristics (**Figure 3**). Strains S4054 and S4054-2 produced the same compounds, but the ratios between the individual compounds were very different in all three strains (**Figures 2A,B**). Precise quantification showed that the concentration of indolmycin increased for both wild type and mutant strains, but that the final concentration in the *maeA* mutant was elevated 3.8-fold when compared to the level in the parental strain S4054-2. A stoichiometric calculation shows that the molar increase in indolmycin production is roughly 1.5-fold higher than the decrease in violacein production (1.2, 1.6, and 1.5, for 24, 48, and 72 h of growth, respectively), suggesting that the metabolism could have been rerouted in the *maeA* mutant.

### Antibacterial Activity of Culture Supernatants from Wild Type and *maeA* Mutant

To test for the antibiotic activity of the three *P. luteoviolacea* strains, cell free extracts were produced to assay their inhibitory effect on the growth of *Vibrio anguillarum* and *Staphylococcus aureus*. Zones of 26-28 mm were found for *V. anguillarum* from 72 h-cultures while 31–32 mm inhibition zones were found for *S. aureus* (**Table 2**). Thus, the difference in violacein concentration in the cultures was not reflected in the size of the inhibition zones. MIC assays confirmed that the culture supernatants had identical levels of antibacterial activity with minimal inhibitory concentration at 32-fold and 128-fold dilutions against *V. anguillarum* and *S. aureus*, respectively (data not shown).

### Inhibition of *Vibrio anguillarum* by Co-culturing with *P. luteoviolacea* Wild Type and Mutant Strains

**FIGURE 4:**
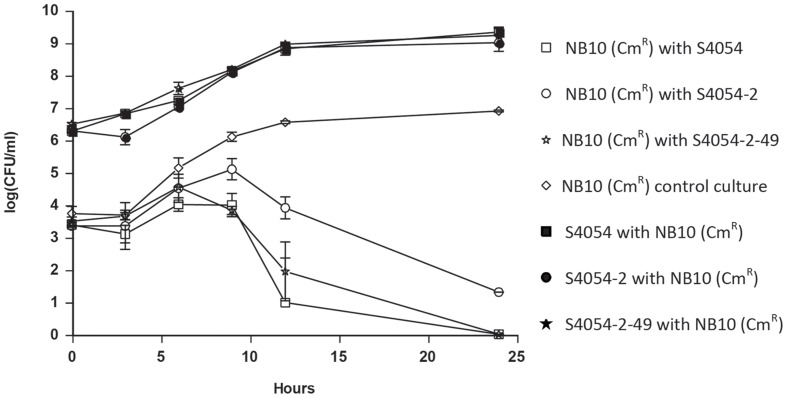
**Co-cultivation of *P. luteoviolacea* strains with *Vibrio anguillarum* NB10 (Cm^R^).** Colony forming units (CFU/ml) of *V. anguillarum* NB10 (Cm^R^) (open symbols) after co-cultivation with S4054 wt (violacein, indolmycin), S4054-2 (violacein, indolmycin) or S4054-2-49 (violacein, indolmycin) as well as CFU/ ml of *P. luteoviolacea* S4054, S4054-2 or S4054-2-49 (black symbol) after co-cultivation with *V. anguillarum* NB10 (Cm^R^) from 0 to 24 h. *Vibrio anguillarum* NB10 (Cm^R^) control culture CFU/ml was also recorded during growth over 24 h.

When *V. anguillarum* NB10 (Cm^R^) was inoculated with S4054, S4054-2, or S4054-2 *maeA* in MMM CAA mannose at a ratio of approximately 1:1000 (10^3^ and 10^6^ cells ml^-1^ for *V. anguillarum* and *P. luteoviolacea*, respectively) the *Vibrio* strain was outcompeted after 25 h of growth (**Figure 4**). When *V. anguillarum* was grown alone, it showed a short exponential growth phase after a lag phase of 3 h, and grew to a cell density of almost 10^7^ CFU ml^-1^. Each of the three *P. luteoviolacea* strains had a similar growth pattern and grew to approximately 10^9^ CFU ml^-1^ by 24 h (**Figure 4**). In co-cultures with *Pseudoalteromonas*, the *V. anguillarum* strains initially grew as in monocultures, but after 6 h where the *P. luteoviolacea* stains had grown to approximately 10^7^ CFU ml^-1^, the number of colony forming *V. anguillarum* cells started to level off. After 12 h where *P. luteoviolacea* entered stationary phase, most *V. anguillarum* cells had died, or were unable to form colonies. The bactericidal effect observed was not dependent on the production of violacein as there was no significant difference in *V. anguillarum* NB10 (Cm^R^) cell numbers when grown together with S4054 (*P* > 0.05) or S4054-2-49 (*P* > 0.05).

## Discussion

*Pseudoalteromonas luteoviolacea* strain S4054 produces both violacein and indolmycin (Månsson et al., 2010). To the best of our knowledge, this is the only reported bacterial species that produce both violacein and indolmycin, leading us to investigate whether the synthesis of the two L-tryptophan derived bioactive compounds were interconnected. Transposon mutagenesis resulted in the isolation of a beige *maeA*::miniTn*10* mutant strain that exhibited a combination of reduced violacein and increased indolmycin production (**Table 2**). Culture supernatants from the *maeA* mutant, grown for 72 h, contained less than 10% of the violacein level present in culture supernatants from the wild type strain S4054 (**Table 2**). Konzen et al. (2006) proposed that violacein is located in the cell membrane of *C. violaceum* to protect the bacterial cell from oxidative stress. This function correlate well with our finding that violacein was only produced in the *maeA* mutant under high oxygen tensions. It is possible that the beige phenotype originating from the decreased violacein production in the mutant made it possible to detect stress induction of violacein genes by their pigmentation, while this induction would normally be hidden by the un-induced violacein production.

**FIGURE 5:**
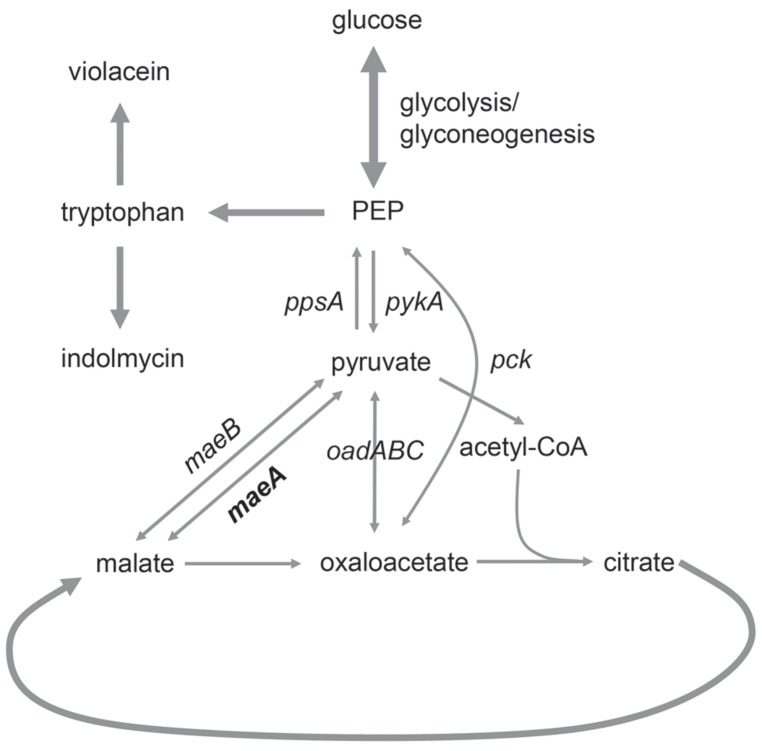
**Malic enzyme MaeA is catalyzing the conversion of malate into pyruvate, which is subsequently used for the production of tryptophan used for violacein and indolmycin biosynthesis**.

The malic enzyme, encoded by the *maeA* gene, catalyzes the conversion of malate into pyruvate and CO_2_ (Kwon et al., 2007). When *P. luteoviolacea* grows on substrates that enter through the citric acid (TCA) cycle, its obligate respiratory energy metabolism is dependent upon replenishing reactions that can provide acetyl-CoA for the continuation of the cycle (**Figure 5**). According to the KEGG database (Kanehisa and Goto, 2000; Kanehisa et al., 2016), four enzymes are able to provide this function in *Pseudoalteromonas* species (data not shown): The malic enzymes MaeA and MaeB require NAD^+^ or NADP^+^ for reduction of malate to pyruvate and CO_2_, respectively, while the phosphoenolpyruvate (PEP) carboxykinase (Pck) and the oxaloacetate decarboxylase (OadABC) are not redox enzymes. The replenishing enzymes are also needed for fueling the gluconeogenesis reactions needed for synthesis of amino acids, nucleotides, lipids, etc. Tryptophan synthesis which is needed for synthesis of both violacein and indolmycin is likely to be affected by the replenishment reactions as two molecules of PEP are consumed for every tryptophan molecule formed, in the AroH and AroA reactions, respectively (Kedar et al., 2007). Disruption of the *maeA* gene would prevent the use of NAD^+^ to replenish pyruvate from malate and the mutant would rely on the remaining three reactions. Furthermore, the conversion of malate into pyruvate is the least thermodynamically favored reaction, but is the one that occurs in nature since the kinetic parameters of the malic enzyme have evolved to favor this direction (Murai et al., 1971; Stols and Donnelly, 1997). However, the opposite direction is actually thermodynamically favored (Goldberg et al., 1993), so with the MaeA encoding gene knocked out, pyruvate is converted into malate without the opposite reaction occurring. Violacein synthesis requires two molecules of tryptophan while indolmycin synthesis requires only one. Therefore the shift from violacein to indolmycin production would suggest that the *maeA* mutation had resulted in decreased tryptophan or PEP production. This appears highly illogical, so our hypothesis is that the conditional production of violacein in the *maeA* mutant is due to regulatory mechanisms controlling the replenishing reactions. Availability of redox co-enzymes, e.g., regeneration of NAD^+^ in the respiratory chain, could also be important, as efficient aeration has been shown to be important for violacein production (Yang et al., 2007). At present the knowledge about these systems in *Pseudoalteromonas* species is too limited to make any conclusions about the exact nature of the cause.

With regard to their antimicrobial activity, culture supernatants from either wild type or *maeA* mutant inhibited the growth of Gram positive *S. aureus* and Gram negative *V. anguillarum* equally well (**Table 2**) in spite of the reduced violacein content in the *maeA* mutant. Since neither violacein nor indolmycin concentration correlated with the size of the inhibition zone, and since violacein is located in the cell membrane and therefore would not be present in our cell-free supernatants, we suggest that a yet unidentified compound could be responsible for the major part of the antimicrobial activity. This hypothesis is supported by the fact that the genome of *P. luteoviolacea* S4054 contains several putative biosynthetic gene clusters, most of which have not yet been assigned a specific function (Machado et al., 2015; Månsson et al., 2016). Out of a total of 36 putative antibiotic and secondary metabolite gene clusters determined by AntiSMASH (Medema et al., 2011; Weber et al., 2015), nine can be attributed a specific function, and a further nine can be attributed a hypothetical function, including production of indolmycin, and the remaining 18 can only be classified on a putative type of gene cluster level, primarily identified by the ClusterFinder algorithm (Weber et al., 2015)

## Author Contributions

MD and LG conceived the research, MD carried out transposon mutagenesis, plate inhibition assays, and co-culture inhibitions, MT and JM carried out MIC assays, MT, JM, and MK carried out substrate-dependent expression, MM and KN performed purification and quantification, BB, CS, and JO performed genome sequencing, assembly and annotation, MT carried out genome analysis, MT, MD, MK, and LG drafted the manuscript. All authors have read and approved the manuscript.

## Conflict of Interest Statement

The authors declare that the research was conducted in the absence of any commercial or financial relationships that could be construed as a potential conflict of interest.

## Footnotes

1 http://blast.ncbi.nlm.nih.gov/Blast.cgi
